# Identifying key variables in African American adherence to colorectal cancer screening: the application of data mining

**DOI:** 10.1186/1471-2458-14-1173

**Published:** 2014-11-18

**Authors:** Vetta L Sanders Thompson, Sean Lander, Shuyu Xu, Chi-Ren Shyu

**Affiliations:** Brown School, Washington University in St. Louis, St. Louis, MO USA; Informatics Institute, University of Missouri, Columbia, MO USA

**Keywords:** Cancer screening, Colorectal cancer, African American

## Abstract

**Background:**

This paper reports on an effort to identify a streamlined set of issues important for colorectal cancer communication and interventions with older African Americans.

**Methods:**

African American (N = 1,021), 683 women and 338 men, 50 to 75 years completed a telephone survey addressing demographics, colorectal cancer screening, cancer attitudes, and cancer related cultural attitudes. Several data analytics methods were applied and evaluated. Among them, results from associative data mining identified key variables and logistic regression was used to confirm associations to screening adherence.

**Results:**

Sets of co-occurring variables identified by associative data mining methods are extracted to further study differences between adherent and non-adherent groups. Logistic regressions suggested four variables were significantly associated with adherence: healthcare provider colonoscopy recommendation, prevention services at the place health care is usually sought, a history of colitis, and a history of polyps.

**Conclusions:**

The findings suggest a streamlined set of issues and concerns that may be used by providers advising patients or developing colorectal cancer intervention strategies for older African Americans. The data suggest the continued importance of healthcare provider recommendation to screen. It is important that providers give a clear recommendation to screen regardless of the test ultimately selected and should advise all patients that family history and the absence of symptoms or colitis do not eliminate the value of screening.

**Electronic supplementary material:**

The online version of this article (doi:10.1186/1471-2458-14-1173) contains supplementary material, which is available to authorized users.

## Background

Colorectal cancer (CRC) is the third most common cancer in both men and women [1] and is also the third most common cause of cancer death among African American men and women [1, 2] in the USA. When detected in early stages, CRC is highly treatable [2] and regular screening facilitates earlier detection, lowers mortality [1–3], and may reduce incidence through removal of pre-cancerous polyps [4, 5]. It is estimated that deaths from CRC could be cut by approximately 60% if all people aged 50 years or older received regular screening tests [6]. Current American guidelines recommend that men and women, ages 50 to 75, screen via one of three methods: an annual fecal occult blood test (FOBT), a sigmoidoscopy (Sig), a combination of annual FOBT and Sig every 5 years, or a colonoscopy (Col) every 7 to 10 years [7].

According to the American Cancer Society, while CRC incidence rates have decreased due to increased use of colorectal cancer screening (CRCS) tests that permit detection and removal of polyps [1], CRC incidence among African American men and women is approximately 20% higher and mortality rates about 45% higher than those among whites [2]. The American Cancer Society states that from 2004 to 2008, annual declines in CRC incidence among white men were much larger than those noted among African American men, 2.9% versus 0.8%, respectively; while among women, declines in CRC incidence among whites (2.2% per year) and African Americans (1.7% per year) were similar [1]. CRC disparities may be partly attributable to differences in African Americans’ screening utilization, which has been linked to later stage of CRC diagnosis among African Americans [1–3]. African American screening prevalence remains less than whites. Furthermore, African CRCS failed to meet the national objective of 50% established by Healthy People 2010 [8] and African American men are known to have lower screening rates than African American women. Thus, CRCS is an underutilized tool given the higher CRC incidence and mortality rates among African Americans [4, 5].

There is a strong need to understand the combination of attitudes and structural factors that result in African American non-adherence to CRC screening guidelines. CRC researchers have focused on a number of variables to understand CRCS adherence, including physician recommendation, usual source of care, cancer worry, perceived risk, benefits and barriers to screening, social norms for CRC screening, CRC screening efficacy, and cultural attitudes relevant to cancer and CRCS [9, 10]. Because of the number of variables, the time required to administer lengthy surveys or query the attitudes found to be relevant to screening decisions are not practical in practice settings. However, strategies have emerged that may permit identification of itemsets, collections of variables, that are relevant for particular populations and are easily used in practice settings. For example, it may be that it is important for health professionals to acknowledge privacy concerns, CRCS concerns such as pain, and discuss family history to encourage CRCS adherence among African American men, but only necessary to discuss family history and give a recommendation to African American women.

As the number of potential variables increases, the number of all possible variable combinations that might explain CRCS adherence and non-adherence will grow exponentially. It is difficult to include all of these candidate combinations in a single statistical model. This is especially true when the research question exhausts theory and becomes non-hypothesis driven; establishing a statistical model for testing becomes time consuming. Therefore, in recent decades, data mining techniques [11] have been applied to many studies in order to discover hidden knowledge based on associations culled from large datasets. Depending on the types of data available, different data mining techniques have been used, such as associative mining [12], temporal mining [13], spatial mining [14], etc. This paper reports on the use of an associative data mining approach [15] to reveal evidence-based associations between combinations of variables and different outcome groups: African American CRCS adherent and non-adherent participants. A suite of data analytic methods from Scikit-learn [16]; such as decision tree [17], support vector machine (SVM) [18], and random forest [19] were applied in addition to associative mining. These methods and their appropriateness for clinical practice are discussed in the Additional file 1. In this research we chose an associative mining approach to evaluate possible strategies that are explainable and implementable.

## Methods

### Participants

African Americans (N = 1,021), 683 women and 338 men, were recruited (2009–2010) to complete a telephone survey. Calls were made using a targeted list sample, created using random digit dial (RDD) generated lists matched to a market research data sample and developed to assure that major geographical regions were represented. In addition to this list, a separate RDD list was purchased and used in calling to reduce biases produced by a listed sample. The samples were drawn by Info USA, which is a company that specializes in developing targeted list samples for low-incidence populations. Eligibility criteria for participation included birth in the United States, self-identified African American male or female aged 50 to 75, a mailing address (for mailing of incentives), and a working telephone number completed a telephone survey.

### Procedures

**Figure 1:**
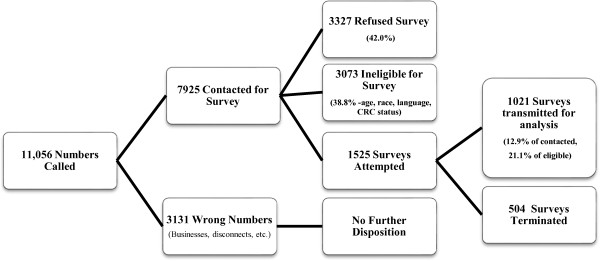
**Survey recruitment statistics.**

The Washington University in St. Louis Institutional Review Board approved this study and the consent procedures used. Listed individuals were contacted by phone via call center. Battelle Centers for Public Health Research and Evaluation trained callers, completed calling and survey administration. Telephone recruiters stated that researchers were recruiting participants for a study of attitudes that may relate to cancer screening, explained eligibility criteria, described the project, and encouraged eligible men and women to participate. If two eligible individuals resided at the residence associated with the telephone number, the Computer Assisted Telephone Interview (CATI) system used a pre-selected random number for the sampled household to determine the respondent. If more than two eligible individuals lived in the household, the most recent birthday, determined who was selected as the respondent and if the respondent was unable to give the birthday, first names were used to determine which eligible adult to select. Figure 1 provides a flow chart of the final study population.

Following eligibility screening, participants provided verbal consent and the survey was administered, which included CRCS and attitude items, cultural variables, and demographic information. The survey took approximately 35 minutes to complete by telephone. Five percent of participants (n = 50) were asked to consent to a re-administration necessary to establish test-retest reliability. Participants in the test-retest group received the follow-up call two weeks after completing the survey.

### Measures

The NCI Self-Report Measure of CRCS was used to assess CRC screening behavior and family history [20]. Experience with three screening tests is assessed: fecal occult blood test (FOBT), sigmoidoscopy (SIG), and colonoscopy [21–24]. Current concordance estimates were all ≥ .80, as were sensitivity and specificity estimates [25]. Kappa statistics for FOBT and SIG were 0.71 and 0.73, respectively. The agreement for COL was almost perfect, 0.89, using Landis & Koch (1977) criteria [26]. Adherence to colorectal cancer screening was determined by classifying those who reported CRC screening by FOBT only within the last year, or SIG within the last five years, SIG within the last five years and FOBT in the last year, those reporting COL within the last seven to ten years (the actual screening interval may be adjusted based on the individual’s CRC risks) were coded as adherent (1); all others were coded as non-adherent (0). The classification criteria are consistent with US Preventive Services Task Force guidelines for CRCS [7].

McQueen’s 8-item perceived pros scale (alpha = 0.75) and 10-item cons scale (alpha = 0.78) were administered [27]. These measures were developed to measure participant perceptions of CRCS pros and cons and have been shown to be invariant across gender, race, and prior CRCS. Responses ranged from not important to very important. The pros and cons items provide information on the attitudes toward CRC and CRC screening among African Americans identified as an important component of TRA.

A 3-item validated scale to measure absolute perceived risk of CRC was administered [9]. Responses range from strongly disagree to strongly agree. In prior studies, the coefficient alpha was 0.79 in male auto-workers and 0.65 in a sample of black and white primary care patients. In addition to this scale, we included an item to assess participants’ comparative perceived risk relative to others their age and sex, which has been shown to be independent of, but positively associated with, absolute perceived risk for CRC.

Based upon TRA/TPB [28], an individual’s subjective norms reflect his/her beliefs about whether or not important referents approve or disapprove of the behavior and would encourage or discourage him/her to engage in CRCS, as well as motivation to comply with those referents. A positive association between subjective norms and engaging in CRCS has been noted [27]. A validated 4-item measure developed specifically for CRCS was administered to assess family and friends’ influence on CRCS [20]. Responses range from 1 = strongly disagree to 5 = strongly agree. Prior studies reported a coefficient alpha of 0.58 in white male auto workers and 0.61 in a clinic sample.

Individuals who feel confident in their ability to perform the required actions to complete CRCS are better able to overcome barriers and get CRCS. A validated 4-item measure of CRCS self-efficacy (alpha = 0.82) was administered [20]. Response options range from strongly disagree to strongly agree or not at all confident to very confident.

Cultural items addressed medical mistrust, fatalism, religiosity, spirituality, collectivism, communalism, racial and ethnic identity, and privacy. *Religiosity*/*spirituality* items addressed the internal manifestation of belief in a higher power and commitment to attendant values [29, 30]. *Fatalism* items focused on the belief that events are beyond an individual’s control [31]. In cancer research, *cancer fatalism*[32, 33] is defined as the belief that death is inevitable when cancer is present. *Racial identification* items referred to a psychological attachment to one of several social categories available to individuals, when the category selected is based on “race” or skin color, common history, nationality, culture, and ancestry [34]. Items covered the centrality, salience, and public and private regard of ethnic identity [35] and racial pride is an aspect of racial identification [36, 37]. *Trust of the medical profession* items addressed the belief that individuals and institutions will act appropriately and in a manner consistent with patients’ interests and included behavioral factors, such as the experience of discrimination [38]. Finally, *collectivism* items assessed the belief that one is linked with family and similar others and hold a cooperative attitude often leading to personal goals being subordinated to those of the group [39].

Data on age, education, income, occupational status and category, and marital status were collected. Items that addressed access to health care and usual source of care were taken from the 2005 National Health Interview Survey, Adult Access to Health Care and Utilization [40].

### Statistical methods/analyses

Descriptive statistics (SPSS, version 17.0, Chicago, IL) were computed to describe the sample and provide scale means and standard deviations.

An associative data mining algorithm [15] was applied in order to explore unknown and potentially relevant relationships among variables found in this dataset. Before using the data mining algorithm, we first divided the dataset into two classes: *C*_*1*_: adherence (*n*_*1*_ = 608); *C*_*2*_: non-adherence (*n*_*2*_ = 411). Each option to one question is treated as an independent and distinct item. For example, if there are four options to one question (*Q*^*1*^), then four items (*i*_*1*_, *i*_*2*_, *i*_*3*_, *i*_*4*_) with a distinct identification code are generated for *Q*^*1*^. Therefore, after this pre-processing, each participant record is represented by a set of disjoint coded items. The complete list of items is considered as candidate variables, which, later on, are used as the input of this associative mining algorithm.

The first step of this algorithm is to identify all frequent itemsets for each group, which are the combination of disjoint items, by calculating support values. Based on the number of unique items in each dataset, there are around 5 choices per question (720 unique items, 144 questions). If an exhaustive approach is applied to search all possible itemsets, (*5*^*144*^) combinations could be generated as potential frequent itemsets. We applied the traditional Apriori algorithm [41] on a Hadoop [42] cluster using Spark [43] to streamline the frequent itemset extraction process. The support threshold for each group is set to 0.6, meaning each discovered frequent itemset had occurred in at least 60% of participant records in each adherent or non-adherent group. This value was empirically chosen after multiple runs of the data with varying support on intervals of 10%. Supports below 60% generated too many itemsets, which are indicative of the population as a whole (non-descriptive). In contrast, supports above 60% filtered valuable discoveries. The support value of itemset (*i*_*1*_, *i*_*2*_) is defined as:

Once the itemset is frequent, the algorithm will start to calculate the confidence value in order to decide whether it is a significant association rule R for a specific adherent group: {*i*_*1*_, *i*_*2*_} → *C*_*1*_:

The frequent itemsets were then filtered so that only maximum supersets remained. In order to find class-specific rules, two methods were used to find itemsets which could be used in a clinical setting, both of which are based on contrast set mining [44]:

1. M1: identifying frequent itemsets that are shared by both adherent groups (*C*_*1*_ and *C*_*2*_) with a significant support difference (at least 20%) between the two groups. {*Itemsets*(*M1*) | *S*_*k*_ ı (*Itemsets* (*C*_*1*_) ∩ *Itemsets* (*C*_*2*_)), where |Support (*S*_*k*_,C1)- Support (*S*_*k*_,C2)| ≥20%}.
2. M2: identifying items (*Ii*’*s*) that are part of a frequent itemset *S*_*k*,*C1*_ = {(*I*_*i*_’s) (*I*_*j*_’s)} in only one of the adherent groups (*C*_*1*_) and a subset of the frequent itemset (*I*_*j*_’s) is also a frequent itemset in another adherence group (C2). This method assists us to find attributes which are strongly shared between groups, but when extra attributes are added, it becomes skewed towards one class or the other.

The findings from both methods were then fed into SPSS (17.0) to perform logistic regression analyses to ensure statistical significance.

## Results

The demographic characteristics of the sample are presented in Table 1. Two participants were excluded due to missing outcome data, resulting in 1019 participants, 681 women and 338 men. Most participants were divorced/separated (42.1%) or currently married/living with a partner (40.1%). The mean age was about 63 years, with a mean of 63.4 years for women and 62.4 years for men. The majority of the sample was highly educated (30.7%), having completed college or a graduate degree; an additional 25.5% had completed some college. Educational attainment was nearly the same for men and women, except among those with some college. The majority of participants had incomes between $10,000 and $74,999, with 37.4% reporting incomes between $10 -$34,999 and 36.7% reporting incomes between $35,000- $74,999. An overwhelming majority of participants reported having insurance, (women, n = 667; 97.6% and men, n = 320; 94.7%). The overall rate of CRCS adherence in this sample was 59.67%.

**Table 1:** **Demographics of the study population by gender** (**N** = **1021**)

Demographics	Overall = 1,021	Male = ***338***	Female = ***683***
Age, mean (SD)	63.1 (7.6)	62.4 (7.5)	63.4 (7.7)
% 50-63	51.4	54.4	49.9
% 64-76	48.6	45.6	50.1
**Education**, %			
Less than High School	3.3	3.3	3.4
Some High School	8.4	8.3	8.5
High School/General Education Diploma	26.5	27.2	26.2
Trade/technical/training school	5.3	4.7	5.6
Some college (no degree)	25.5	22.2	27.1
College degree	18.3	21.3	16.8
Graduate degree	12.4	12.4	12.4
Refused	0.2	0.6	
**Income**, %			
<$10,000	8.8	7.4	9.5
$10,000-$19,999	18.0	14.8	19.6
$20,000-$34,999	19.4	16.9	20.6
$35,000-$49,999	14.7	15.1	14.5
$50,000-$74,999	12.0	13.0	11.6
$75,000-$99,999	7.6	10.4	6.3
>$100,000	5.6	10.1	3.4
Refused	9.1	8.0	9.7
Not sure/Don’t know	4.7		4.8
**Employed**			
No	71.5	66.3	74.1
Part-time	8.1	6.5	8.9
Full-time	20.2	26.9	16.8
Refused	0.2	0.3	0.1
**Marital status**, %			
Single	17.5	17.2	17.7
Married/partnered	40.1	51.8	34.3
Divorced/separated/widowed	42.1	30.5	47.9
Refused	0.3	0.6	0.1

### Associative mining findings

The use of an associative mining algorithm resulted in identification of the following relevant itemsets (shown in Tables 2, 3, and 4). The largest itemset with high support for adherence (*n*_*1*_ = 608) contained six variables, while the largest itemset with high support for non-adherence (*n*_*2*_ = 411) contained five variables.

**Table 2:** **Sample from findings of strong adherent itemsets**

Itemset 1	Itemset 2 adherent	Itemset 3 adherent
3001: # of telephones in household [1]		
130001: Is there a place that you USUALLY go to when you are sick or need advice about your health? [Yes]		
133001: Is that the same place you USUALLY go when you need routine or preventative care, such as a physical examination or check up? [Yes]		
137001: When I don’t know something, I don’t at all mind admitting it. [Yes]		
139001: I would never think of letting someone else be punished for my wrong-doings [Yes]		
	68002: Was either of your parents or any of your brothers or sisters ever diagnosed with colorectal cancer? [No]	
	132001: What kind of place do you go most often, a clinic, doctor’s office, emergency room, or some other place? [Clinic or Health center]	
	139001: I would never think of letting someone else be punished for my wrong-doings [Yes]	
		67004: Being too embarrassed to talk to your doctor about colon cancer. [Not important]
		132001: What kind of place do you go most often, a clinic, doctor’s office, emergency room, or some other place? [Clinic or Health center]
		139001: I would never think of letting someone else be punished for my wrong-doings [Yes]

Itemsets which had at least 80% support from the Adherence Group and did not appear in the Non-adherent Group with support above 60%.

**Table 3:** **Sample from findings of strong non**-**adherent itemsets**

Itemset 1 non-adherent	Itemset 2 non-adherent
31002: Has a doctor ever told you that you had Chron’s, Colitis, IBS [No]	
68002: Was either of your parents or any of your brothers or sisters ever diagnosed with colorectal cancer? [No]	
	31002: Has a doctor ever told you that you had Chron’s, Colitis, IBS [No]
	133001: Is that the same place you USUALLY go when you need routine or preventative care, such as a physical examination or check up? [Yes]

Itemsets which had at least 80% support from the Non-adherence Group and did not appear in the Adherence Group with support above 60%.

**Table 4:** **Sample from findings of strong pairwise subsets and predictive differences for adherence**

Adherent and non-adherent	Adherent only
16001: Before these tests were described, had you ever heard of a colonoscopy? [Yes]	18001: Have you ever had a colonoscopy? [Yes]
130001: Is there a place that you USUALLY go to when you are sick or need advice about your health? [Yes]	133001: Is that the same place you USUALLY go when you need routine or preventative care, such as a physical examination or check up? [Yes]
16001: Before these tests were described, had you ever heard of a colonoscopy? [Yes]	18001: Have you ever had a colonoscopy? [Yes]
130001: Is there a place that you USUALLY go to when you are sick or need advice about your health? [Yes]	139001: I would never think of letting someone else be punished for my wrong-doings [Yes]
16001: Before these tests were described, had you ever heard of a colonoscopy? [Yes]	18001: Have you ever had a colonoscopy? [Yes]
139001: I would never think of letting someone else be punished for my wrong-doings [Yes]	130001: Is there a place that you USUALLY go to when you are sick or need advice about your health? [Yes]
16001: Before these tests were described, had you ever heard of a colonoscopy? [Yes]	17001: Did a doctor, nurse, or other health professional ever advise you to get a colonoscopy? [Yes]
132001: What kind of place do you go most often, a clinic, doctor’s office, emergency room, or some other place? [Clinic or Health center]	
137001: When I don’t know something, I don’t at all mind admitting it. [Yes]	
16001: Before these tests were described, had you ever heard of a colonoscopy? [Yes]	17001: Did a doctor, nurse, or other health professional ever advise you to get a colonoscopy? [Yes]
132001: What kind of place do you go most often, a clinic, doctor’s office, emergency room, or some other place? [Clinic or Health center]	
139001: I would never think of letting someone else be punished for my wrong-doings [Yes]	

Itemsets which appear on the left appear in both adherence groups. Items on the right, when added to the itemset on the left, appear only in the Adherent Group.

In Tables 2 and 3, the cells represent itemsets from the survey which have a high support for one group, but have sub-60% support for the other. For example, there was a high occurrence of those in the adherence group not being embarrassed about talking with their doctor about colon cancer, going to a clinic when they get sick, and not considering letting someone be punished for their wrong-doings. This combination appeared 80% of the time among those in the adherence group, but appeared less than 60% of the time in the non-adherence group. Subsets of this may appear with higher support among non-adherents, but the unique combination is specific to those who are adherent to CRCS.

In Table 4, the cells on the left represent a subset which appears in high support itemsets from both groups. The cell on the right represents the itemset to be added to the left which makes it unique to the Adherent Group. These supersets have a sub-60% support in the non-adherence group, lending them significance when they exist together. The itemset on the right, therefore, has high predictive power towards one group or the other, depending on whether it exists in a new record.

Surprisingly, there were no usable itemsets for non-adherence when using pairwise subsets, while the largest itemset from the adherence pairwise subsets contained seven variables, three of which did not appear in the largest non-adherent subset.

The issues identified represent two categories - access and attitudes, which can be addressed when developing colorectal cancer health communication and interventions strategies for this population. Those related to adherence focused on physician recommendation and personal disease risk factors. The itemsets related to non-adherence were focused on awareness of a family history of disease and the actual presence of polyps or colitis. Items that addressed attempts to achieve a positive self-presentation were included in the survey on CRCS and were included in the final item sets.

### Logistic regression

Physician or healthcare colonoscopy recommendation, history of colitis, history of polyps, family history of CRC, usual source of health care, receipt of prevention services at the place health care is usually sought, feeling embarrassed discussing CRCS with a doctor; and two items that suggest impression management – I would never allow others to be punished for something that I did and I will admit when I do not know the answer to a question - were included as independent variables in the logistic regression. The item addressing the number of phones in the home was omitted. The full model is presented in Table 5. Four items classify individuals who are CRCS adherent (53.4% of non-adherent and 83.6% of adherent participants): physician or healthcare provider colonoscopy recommendation, receipt of prevention services at the place health care is usually sought and a history of colitis or polyps (Cox & Snell R^2^ = .14, Nagelkerke R^2^ = .19; Hosmer and Lemeshow *χ*^2^ (5) = 4.42, p = .49). Individuals with no physician recommendation to receive colonoscopy, who had not received preventive health services at the place they usually sought health care and had no history of colitis or polyps were less likely to be CRCS adherent.

**Table 5:** **Multivariate logistic regression of non**-**adherence vs. adherence with CRCS** (**N** = **911**)

Item	B	S. E. (B)	Wald	OR (95% CI)
Provider recommendation***	1.48	.18	71.48	0.23 (0.16, 0.32)
No history of colitis***	1.51	.33	21.29	4.51 (2.38, 8.55)
Not told of polyps***	.27	.09	8.95	0.77 (0.64,0.91)
Family history	0.07	0.09	0.57	1.07 (.90, 1.28)
Usual source of care	0.07	0.29	0.06	0.93 (0.53, 1.65)
Preventive services at usual place of care***	1.22	0.35	12.15	0.30 (0.15, 0.59)
Embarrassed to talk to doctor	0.04	0.09	0.17	1.04 (0.88, 1.23)
Admit not know	0.05	0.17	0.09	0.95 (0.69, 1.32)
Allow others to be punished for my actions	0.25	0.18	2.00	0.78 (0.55, 1.10)

***p < .001, CI: confidence interval.

## Discussion and conclusion

The findings suggest a streamlined set of issues and concerns that may assist in efforts to improve adherence to CRCS among older African Americans. Itemsets identified using an associative data mining technique include items that are consistent with factors previously identified in the screening adherence literature; health practitioner recommendation to screen, having a risk factor for CRC, and a usual source of care are predicted using the TRA/TPB [9, 10]. Knowledge and health practitioner recommendations help to inform attitudes about the importance and positive consequences of screening; risk factors, such as prior polyps and/or colitis; increase the sense of susceptibility to the disease and reinforce attitudes related to the importance of screening. A usual source of care can be seen as reducing the barriers to screening and may stimulate a sense of screening as a normative behavior. The importance of a physician recommendation for colonoscopy, as indicated by its prominence in itemsets, may signal the importance of physicians as influencers in health decision making [45].

The importance of a usual source of care and the receipt of preventive services through that source of health care highlight the importance of access concerns. The inclusion of the item addressing the number of phones in the home in itemsets may also reflect access issues related to scope based CRCS scheduling and logistics. While not included in the logistic regression, it may suggest the need to determine whether patients have convenient and easy access to the resources required to successfully complete the more complicated process of obtaining a scope based screening test.

The items that were more strongly associated with non-adherence suggest that non-adherent African American participants without risk factors may not have perceived sufficient reason to act or see CRCS as normative behavior. This explanation is supported by the fact that social and cultural variables were unrelated to CRCS adherence. The discussion of CRCS in social and cultural terms may be necessary to generate a perceived need to screen. This issue can be tested and cultural tailoring may be relevant in evaluations of education materials tailored for special populations [46]. Messages for these individuals might also highlight the fact that CRC may occur in the absence of family history and the fact that the presence of polyps is only detected via screening at appropriate intervals after polyps have been detected. It is not surprising that non-adherent individuals have not been told that they have polyps, as identification of polyps is unlikely without endoscopic screening; further, the presence of polyps may shorten screening intervals [4, 5] for adherent individuals who report receiving this information. An alternative explanation of the presence of colitis in the non-adherent itemset may be that these individuals may have regular endoscopic procedures and additional procedures for preventive care may not be needed.

Several items were identified that suggest new concerns that may be important in African American screening adherence. The role of impression management in CRCS is unclear. The failure to select ‘false’ to the item “When I don’t know something, I don’t at all mind admitting it,” is an example worth considering. A plausible explanation for the role of impression management is that patients responding ‘false’ have a hard time admitting flaws and will not ask questions when they do not understand their options or the recommendations made. These patients may be more likely to respond affirmatively to physician recommendations to screen and may not readily report concerns or questions related to screening. Even when CRCS is completed, it does not assure that the patient’s needs are met. For example, CRCS screening prep may not be optimal, which could result in missed polyps. In addition, a patient might incur unexpected costs that might inhibit future CRCS. These and the explanations provided for other findings are speculative and can only be resolved through additional research.

While these data suggest a streamlined set of issues and concerns that may assist in efforts to improve adherence to CRCS among older African Americans, there are limitations to the findings. This study included a wide range of items representing a comprehensive set of issues identified as important to screening in the literature; however the items and constructs included are not exhaustive. For example, we cannot examine how issues of insurance or a usual source of care vary among retired versus unemployed individuals nor aspects of religiosity or social identities not assessed. There are a number of data mining strategies that can be applied and these might have highlighted a different set of issues. CRCS adherence status was determined by self-report and the accuracy of self-reported status may affect the accuracy of the factors associated with adherence and non-adherence. Although earlier studies suggested that the validity of self-reported CRCS was low [21, 22], recent evidence suggests pre-testing and careful revision of survey instruments can result in significant improvements in the validity of self-reports [23, 24]. By including descriptions of the various tests in the assessment instrument and revising the instrument after CRT testing with the population, Baier found that the instrument resulted in highly accurate self-reporting of CRCS tests. Specificity of FOBT recall rose from 64% to 86% [24]. Also, more is known about factors that support adherence than is known about those that drive non-adherence and the constructs addressed may be weighted toward adherence issues. While the findings of this study were completed using a large national sample of African Americans eligible for colorectal cancer screening, it was not representative of a national sample of older African Americans and a representative sample might yield different results.

### Conclusion

While it is important to explore a wide range of variables theoretically linked to CRCS, it is also important to identify a small number of critical variables that can be effectively addressed while advising patients on CRCS. The current findings confirm the importance of three issues currently assessed and examined in the empirical literature [9, 10, 45]; healthcare provider recommendation, a clear understanding of CRCS importance regardless of personal or family history, and access to preventive care [10]. Healthcare providers should determine whether patients recognize that they have been given a recommendation to screen and that screening is recommended regardless of family history or symptoms of disease [4, 5], but may be more important in the presence of a family history of CRC and symptoms of disease. Allied healthcare staff (medical social workers, navigators, lay health advisors, etc.) should assure that patients know where to go for CRCS and receive affordable screening options. Additional research is required to determine whether the importance of a provider recommendation to obtain colonoscopy was more important than other screening recommendations due to patient preference, screening interval or other factors not examined in this paper.

Given the novelty of the impression management findings, it will be important to determine how efforts to be viewed favorably by providers affect patient honesty when there are concerns over screening recommendations. Research is needed to determine if there are specific intervention and education strategies that heighten self- presentation and impression management responses among African American older adults. Finally, researchers should attempt to identify specific health education and promotion strategies that are resistant to an impression management response set.

### Practice implications

The data reported suggest that it is important that healthcare providers give a clear recommendation to screen regardless of the test ultimately selected and should advise all patients that family history and the absence of symptoms or colitis do not eliminate the value of screening [10]. While this suggestion is not new, it emphasizes the importance of highlighting the possibility of disease in the absence of family history or symptoms. Healthcare providers should attend to signals that patients may be embarrassed to discuss issues or are attempting to manage provider impressions of them. It may be important for providers to consider whether patients are attempting to disguise a lack of understanding, fears and concerns about CRCS and their true intent to screen. While impression management items cannot be asked during clinical encounters, there are steps that healthcare providers can take to address self- presentation concerns, such as asking participants about their plans for specific activities related to CRCS prep (time off from work, someone to accompany them to a screening) or feelings about handling stool for an FOBT and their comfort directly discussing the procedure with family members. All evidence based screening options should receive equal attention when presenting options to patients so that those with cost concerns, including issues of managing copays or the costs of preparation prescriptions, are not embarrassed or hesitant to select an affordable option. Health care providers should also assure that they have CRCS materials that are plain language and easy to read.

In addition, the data suggest the importance of cultivating the use of a range of preventive health services prior to the age for the initiation of CRCS. In advising patients about CRCS, particular attention should be paid to patients who have been non-adherent to other medical recommendations, particularly if this behavior was unexpected based on patient statements.

## Electronic supplementary material

Additional file 1:**Results of Test of Data Analytic Methods in Machine Learning and Data Mining.**(PDF 601 KB)

## References

[1] American Cancer Society (2012). Cancer Facts & Figures 2012.

[2] American Cancer Society (2011). Colorectal Cancer Facts & Figures 2011–2013.

[3] Horner MJ, Ries LAG, Krapcho M, Neyman N, Aminou R, Howlader N, Altekruse SF, Feuer EJ, Huang L, Mariotto A, Miller BA, Lewis DR, Eisner MP, Stinchcomb DG, Edwards BK (2009). SEER Cancer Statistics Review, 1975–2006.

[4] Byers T, Levin B, Rothenberger D, Dodd G, Smith R (1997). American Cancer Society Guidelines for screening and surveillance for early detection of colorectal polyps and cancer: update 1997. American cancer society detection and treatment advisory group on colorectal cancer. CA-Cancer J Clin.

[5] National Cancer Institute (2006). What you Need to Know About Cancer of the Colon and Rectum.

[6] *Centers for Disease Control and Prevention. Top 10 Cancers Among Men*. 2012. http://apps.nccd.cdc.gov/uscs/toptencancers.aspx. Retrieved November 24, 2014

[7] U.S. Preventive Services Task Force (2008). Screening for colorectal cancer: U. S. Preventive Services Task Force recommendation. Ann Intern Med.

[8] U. S. Department of Health and Human Services (2000). Healthy People 2010 Objectives.

[9] Thompson Sanders VL, Harris J, Joo S (2011). “Measuring Cultural Attitudes Relevant to Cancer Screening and Behavior”.

[10] Thompson VLS, Kalesan B, Wells A, Williams S-L, Caito N (2010). Comparing the use of evidence and culture in targeted colorectal cancer communication for African Americans. Patient Educ and Couns.

[11] Han J, Kamber M, Pei J (2011). Data Mining: Concepts and Techniques.

[12] Agrawal R, Imieliński T, Swami A (1993). Mining association rules between sets of items in large databases. ACM SIGMOD Record.

[13] Das G, Lin KI, Mannila H, Renganathan G, Smyth P (1998). Rule discovery from time series. KDD Proc.

[14] Miller HJ, Han J (2009). Geographic Data Mining and Knowledge Discovery.

[15] Xu S, Shyu CR (2010). Efficient selection of association rules from lymphedema symptoms data using a graph structure. AMIA Annu Symp Proc.

[16] Pedregosa F, Varoquaux G, Gramfort A, Michel V, Thirion B, Grisel O, Blondel M, Prettenhofer P, Weiss R, Dubourg V, Vanderplas J, Passos A, Cournapeau D, Brucher M, Perrot M, Duchesnay E (2011). Scikit-learn: machine learning in Python. JMLR.

[17] Thomas TM (1997). Machine Learning.

[18] Cortes C, Vapnik V (1995). Support-vector networks. Mach Learn.

[19] Breiman L (2001). “Random Forests”. Mach Learn.

[20] Vernon SW, Myers RE, Tilley BC (1997). Development and validation of an instrument to measure factors related to colorectal cancer screening adherence. Cancer Epidemiol Biomarkers Prev.

[21] Chaiken S, Eagly AH (1976). Communication modality as a determinant of message persuasiveness and message comprehensibility. J Pers Soc Psychol.

[22] Mandelson MT, LaCroix AZ, Anderson LA, Nadel MR, Lee NC (1999). Comparison of self-reported fecal occult blood testing with automated laboratory records among older women in a health maintenance organization. Am J Epidemiol.

[23] Vernon SW, Meissner H, Klabunde C, Rimer BK, Ahnen DJ, Bastani R, Mandelson MT, Nadel MR, Sheinfeld-Gorin S, Zapka J (2004). Measures for ascertaining use of colorectal cancer screening in behavioral, health services, and epidemiologic research. Cancer Epidemiol Biomarkers Prev.

[24] Baier M, Calonge N, Cutter G, McClatchey M, Schoentgen S, Hines S, Marcus A, Ahnen D (2000). Validity of self-reported colorectal cancer screening behavior. Cancer Epidemiol Biomarkers Prev.

[25] Vernon SW, Tiro JA, Vojvodic RW, Coan S, Diamond PM, Greisinger A, Fernandez ME (2008). Reliability and validity of a questionnaire to measure colorectal cancer screening behaviors: Does mode of survey administration matter?. Cancer Epidemiol Biomarkers Prev.

[26] Landis JR, Koch GG (1977). The measurement of observer agreement for categorical data. Biometrics.

[27] McQueen A, Tiro JA, Vernon SW (2008). Construct validity and invariance of four factors associated with colorectal cancer screening across gender, race, and prior screening. Cancer Epidemiol Biomarkers Prev.

[28] Fishbein M, Ajzen I (2010). Predicting and Changing Behavior: The Reasoned Action Approach.

[29] Office of Behavior and Social Science Research (2004). Progress and Promise in Research on Social and Cultural Dimensions of Health: A Research Agenda.

[30] Mattis JS (2000). African American women’s definitions of spirituality and religiosity. J Black Psychol.

[31] Hiatt RA, Pasick RJ, Pérez-Stable EJ, McPhee SJ, Engelstad L, Lee M, Sabogal F, Carol ND, Onofrio SS (2008). Pathways to early cancer detection in the multiethnic population of the San Francisco bay area. Health Educ Behav.

[32] Powe BD (1995). Fatalism among elderly African Americans: effects on colorectal cancer screening. Cancer Nurs.

[33] Powe BD (2001). Cancer fatalism among elderly African American women. J Psychosoc Oncol.

[34] Thompson Sanders VL (1990). Factors affecting the level of African American identification. J Black Psychol.

[35] Sellers R, Smith M, Shelton J, Rowley S, Chavous T (1998). Multidimensional model of racial identity: a reconceptualization of African American racial identity. Pers Soc Psychol Rev.

[36] Resnicow K, Soler RE, Braithwaite RL, Selassie MB, Smith M (1999). Development of a racial and ethnic identity scale for African American adolescents: the survey of black life. J Black Psychol.

[37] Yancey AK, Aneshensel CS, Driscoll AK (2001). The assessment of ethnic identity in a diverse urban youth population. J Black Psychol.

[38] Thompson Sanders VL, Bazile A, Akbar M (2004). (2004). African Americans’ perceptions of psychotherapy and psychotherapists. Prof Psychol-Res Pract.

[39] Jagers RJ, Mock LO (1995). The communalism scale and collectivistic-individualistic tendencies: some preliminary findings. J Black Psychol.

[40] National Health Interview Survey (2005). National Health Interview Survey, Adult Access to Health Care and Utilization, 2007.

[41] Agrawal R, Mannila H, Srikant R, Toivonen H, Verkamo AI (1996). Fast discovery of association rules. Advances in Knowledge Discovery and Data Mining.

[42] Borthakur D (2007). The hadoop distributed file system: architecture and design. Hadoop Proj Website.

[43] Zaharia M, Chowdhury M, Franklin MJ, Shenker S, Stoica I (2010). Spark: cluster computing with working sets. Proceedings of the 2nd USENIX conference on Hot topics in cloud computing.

[44] Bay SD, Pazzani MJ (2001). Detecting group differences: mining contrast sets. Data Min Knowl Disc.

[45] Thompson Sanders V, Cavazos-Rehg P, Rutten LF, Hesse BW, Moser RP, Kreps GL (2010). Health Information National Trends Survey: Implications for Addressing Cancer Health Disparities through Public Health Surveillance. Building the Evidence Base in Cancer Communication: Hints.

[46] Dreier M, Borutta B, Seidel G, Kreusel I, Töppich J, Bitzer EM, Dierks ML, Walter U (2013). Development of a comprehensive list of criteria for evaluating consumer education materials on colorectal cancer screening. BMC Public Health.

[CR47] The pre-publication history for this paper can be accessed here:http://www.biomedcentral.com/1471-2458/14/1173/prepub

