# Supplementary material for: Identifying key variables in African American adherence to colorectal cancer screening: the application of data mining
Source: BMC Public Health. 2014 Nov 18;14:1173. doi: 10.1186/1471-2458-14-1173 (PMC4256804; doi:10.1186/1471-2458-14-1173)

Supplemental Appendix

Overview

Multiple machine learning algorithms were tested before arriving at the use of associative rule mining, including support vector machines (SVMs), decision trees, and random forests. Though the accuracy in prediction was high for some, the usability of the predictors in a clinical setting would be low due to their different failings. All tests were run on three versions of the dataset: the full dataset, a dataset with class-identifying variables removed, and a dataset with all behavior factor variables removed. Class-identifying variables are the three variables used to determine the classes in accordance with the US Preventive Services Task Force guidelines for CRCS. Behavioral factors are those listed in the African American Multi-Cultural Constructs Survey Codebook used during data collection, and will be referenced as clinical variables for our uses, as they are variables whose data may exist in medical records. The three datasets used are labeled as

- Full – all variables
- Noident – WHNCOL, WHNSIG, and WHNFOBT variables removed
- Noclinical – All clinical variables removed

Overall, the classification accuracy of the tree-based approaches performed the best, with non-identifying accuracies of nearly 94% on average for decision trees of depth 5 and 6. Random forest neared 93% given the same circumstances, showing it as a powerful classifier as well, though the simpler decision tree would be preferred. SVMs had the lowest performance overall, with only 74% classification accuracy on the same dataset across all runs.

Support Vector Machine (SVM)

SVM tests were run using ten-fold cross validation across each of the three datasets. SVMs had the lowest performance, even when using the full dataset. Of the kernels used, the radial basis function had the best accuracy. SVMs are difficult to analyze, however, so it was difficult to tell which variable(s) caused the inaccuracy, or indeed even which variables effected the SVM's classification the most. Due to this, SVM was not considered as an appropriate tool for non-adherence classification.

Average of Full	Average of Noident	Average of Noclinical
0.7752	0.7301	0.6369

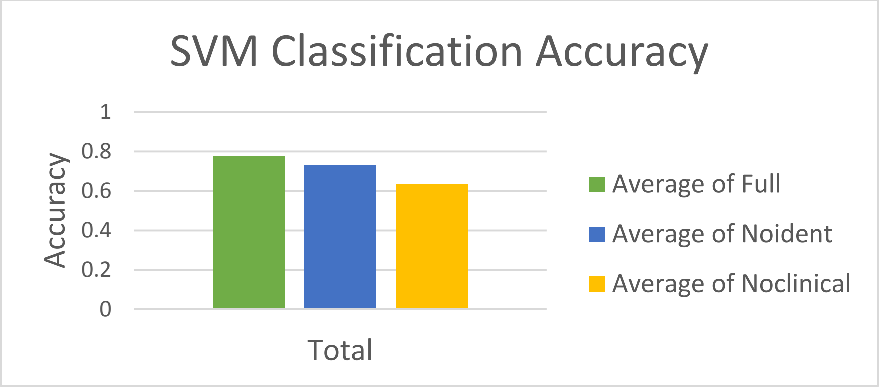

Decision Trees

In the following table, the **bold** number on the left is the number of features allowed to be used during the tree's creation, and the non-bold number to the right of it is the depth of the tree. Just like the SVM, the decision trees were run against all datasets using ten-fold cross validation, with the average accuracy taken for each features, depth combination.

Decision trees had a better prediction accuracy than SVMs overall. Decision trees are normally a good choice in situations like this where a clinician can compare a chart to a tree to make a decision. The depth of the tree and number of features required hampered this, however, with the more accurate trees being deep and varied. This removed it as a good candidate, as much of its prediction power lay in leaf nodes with few data points. In the shallower trees, leaf nodes with large numbers of individuals sometimes contained distributions close to even, while the deep trees became harder to navigate and did not contain populations large enough for confidence in the result. These two factors caused us to not pursue decision trees. An example of one of the trees, with all features and depth of 4, appears at the bottom of this appendix. If decision trees were to be used in this way in the future, a depth of 4 seemed to give good results while maintaining readability.

The drawbacks of the decision tree can be seen in the attached tree, however, such as an individual with whycol < 0.5 and numfobt > 1.5. 48 individuals are classified in this group, yet the next split reduces to 40 and 8. From the 40 samples, it breaks down to groups of 38 and 2 given the recscree score, which means many of the classifications are based on a small number of samples.

Features, Depth	Average of Full	Average of Noident	Average of Noclinical
**5**	**0.72227**	**0.70763**	**0.60628**
2	0.6829	0.6506	0.6114
3	0.6694	0.6812	0.6222
4	0.6918	0.6938	0.6222
5	0.7232	0.732	0.5995
6	0.7361	0.7546	0.5957
7	0.7115	0.7213	0.6104
8	0.7772	0.7173	0.6251

9	0.7497	0.728	0.6026
10	0.7518	0.7281	0.6035
None	0.7291	0.6694	0.5702
**10**	**0.76764**	**0.74141**	**0.60919**
2	0.7116	0.6859	0.6114
3	0.6752	0.6722	0.6193
4	0.7723	0.7448	0.6339
5	0.7713	0.7763	0.6123
6	0.7567	0.8028	0.6055
7	0.8234	0.7282	0.632
8	0.7939	0.737	0.625
9	0.7861	0.7252	0.5898
10	0.79	0.789	0.6024
None	0.7959	0.7527	0.5603
**15**	**0.8005**	**0.79021**	**0.61371**
2	0.7302	0.7155	0.6203
3	0.788	0.7821	0.634
4	0.7988	0.7626	0.6084
5	0.8096	0.8146	0.6172
6	0.8018	0.8058	0.6084
7	0.8165	0.7938	0.6231
8	0.8224	0.8156	0.6231
9	0.8253	0.792	0.6123
10	0.7841	0.8174	0.6123
None	0.8283	0.8027	0.578
**20**	**0.82011**	**0.79649**	**0.61108**
2	0.7959	0.7193	0.6311
3	0.8125	0.7498	0.6359
4	0.8077	0.838	0.6192
5	0.8116	0.7812	0.6271
6	0.8155	0.8018	0.629
7	0.8194	0.8293	0.6094
8	0.8527	0.8106	0.6054
9	0.8302	0.795	0.5829
10	0.8214	0.8214	0.6006
None	0.8342	0.8185	0.5702
**25**	**0.83848**	**0.81658**	**0.61656**
2	0.7949	0.7488	0.6251
3	0.843	0.7812	0.6408
4	0.8234	0.8096	0.6192
5	0.8626	0.8086	0.63
6	0.8557	0.8529	0.6202

7	0.8312	0.8291	0.6379
8	0.84	0.8597	0.6222
9	0.8421	0.8145	0.6143
10	0.8528	0.8351	0.5897
None	0.8391	0.8263	0.5662
**30**	**0.83571**	**0.8312**	**0.61737**
2	0.7831	0.7665	0.6113
3	0.8056	0.8371	0.636
4	0.8371	0.8174	0.6271
5	0.8351	0.8047	0.5869
6	0.8558	0.8341	0.6251
7	0.841	0.8311	0.6261
8	0.841	0.8479	0.6349
9	0.8538	0.8606	0.633
10	0.841	0.8627	0.6065
None	0.8636	0.8499	0.5868
**50**	**0.87045**	**0.86272**	**0.62052**
2	0.7919	0.7851	0.6438
3	0.8685	0.8421	0.6252
4	0.8597	0.8548	0.6262
5	0.8695	0.8881	0.6104
6	0.8901	0.8617	0.6232
7	0.896	0.8822	0.6408
8	0.9037	0.8813	0.6212
9	0.8744	0.8832	0.6026
10	0.8793	0.8851	0.6231
None	0.8714	0.8636	0.5887
**75**	**0.90391**	**0.89489**	**0.62238**
2	0.8352	0.842	0.6409
3	0.8901	0.8763	0.6252
4	0.9009	0.8901	0.6418
5	0.9195	0.9185	0.627
6	0.9303	0.9029	0.6379
7	0.9097	0.9156	0.6359
8	0.9195	0.9038	0.6271
9	0.9214	0.9117	0.6045
10	0.9048	0.8979	0.6084
None	0.9077	0.8901	0.5751
**100**	**0.91804**	**0.90195**	**0.63453**
2	0.8586	0.84	0.6526
3	0.8969	0.895	0.6408
4	0.9215	0.9136	0.6369

5	0.9234	0.8989	0.6418
6	0.9352	0.9166	0.629
7	0.9421	0.9137	0.633
8	0.9274	0.9205	0.6526
9	0.9264	0.9136	0.6281
10	0.9333	0.8979	0.6349
None	0.9156	0.9097	0.5956
**None**	**0.94012**	**0.92062**	**0.63904**
2	0.8989	0.8989	0.6507
3	0.9117	0.893	0.6389
4	0.9362	0.9235	0.6526
5	0.9548	0.9382	0.6555
6	0.9529	0.9392	0.6388
7	0.949	0.9303	0.6427
8	0.9519	0.9264	0.6487
9	0.9499	0.9215	0.6339
10	0.9499	0.9176	0.6379
None	0.946	0.9176	0.5907
**Grand Total**	**0.841723**	**0.82637**	**0.619066**

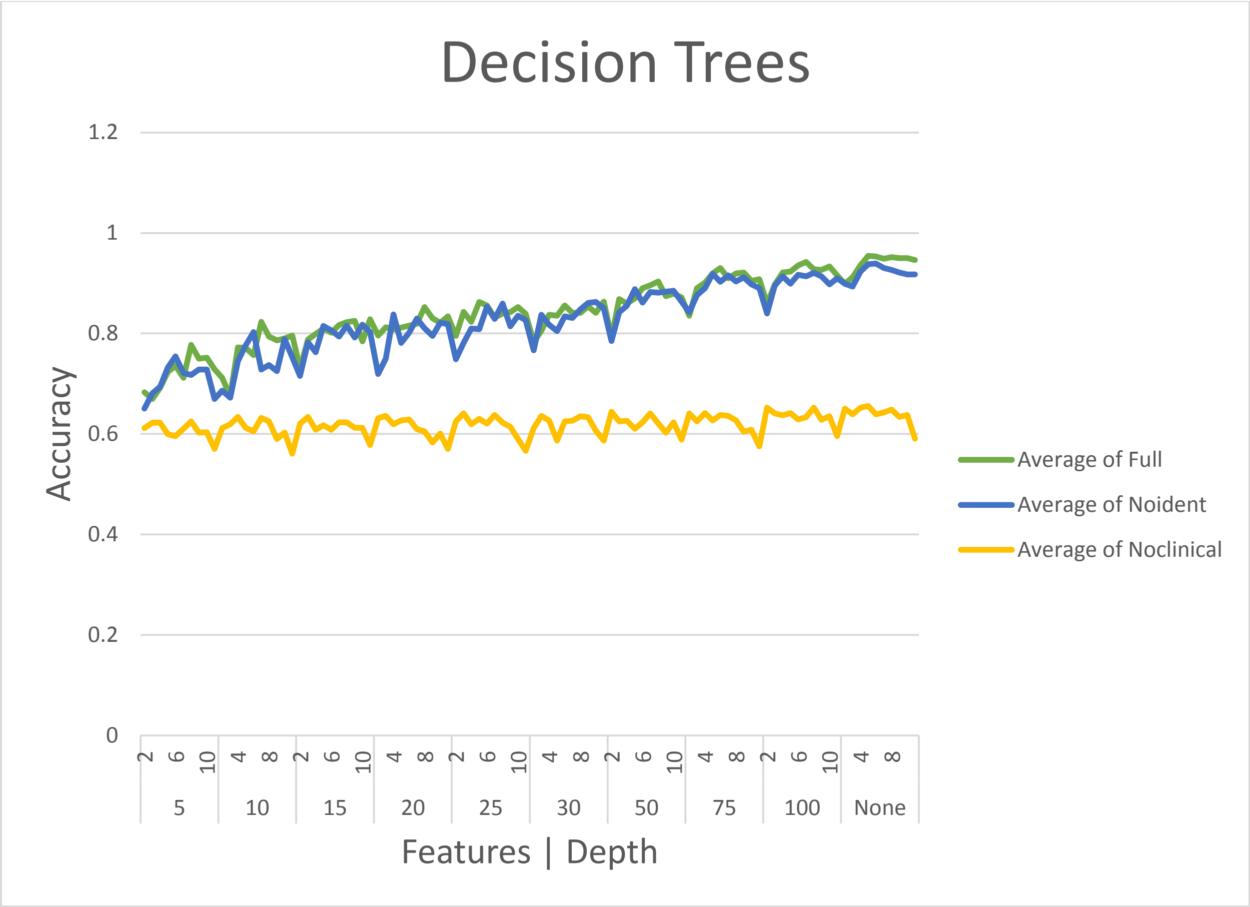
Decision Trees
1.2
1
0.8
0.6
0.4
0.2
0
Accuracy
2 6 10 4 8 2 6 10 4 8 2 6 10 4 8 2 6 10 4 8 2 6 10 4 8
5 10 15 20 25 30 50 75 100 None
Features | Depth
Average of Full
Average of Noident
Average of Noclinical

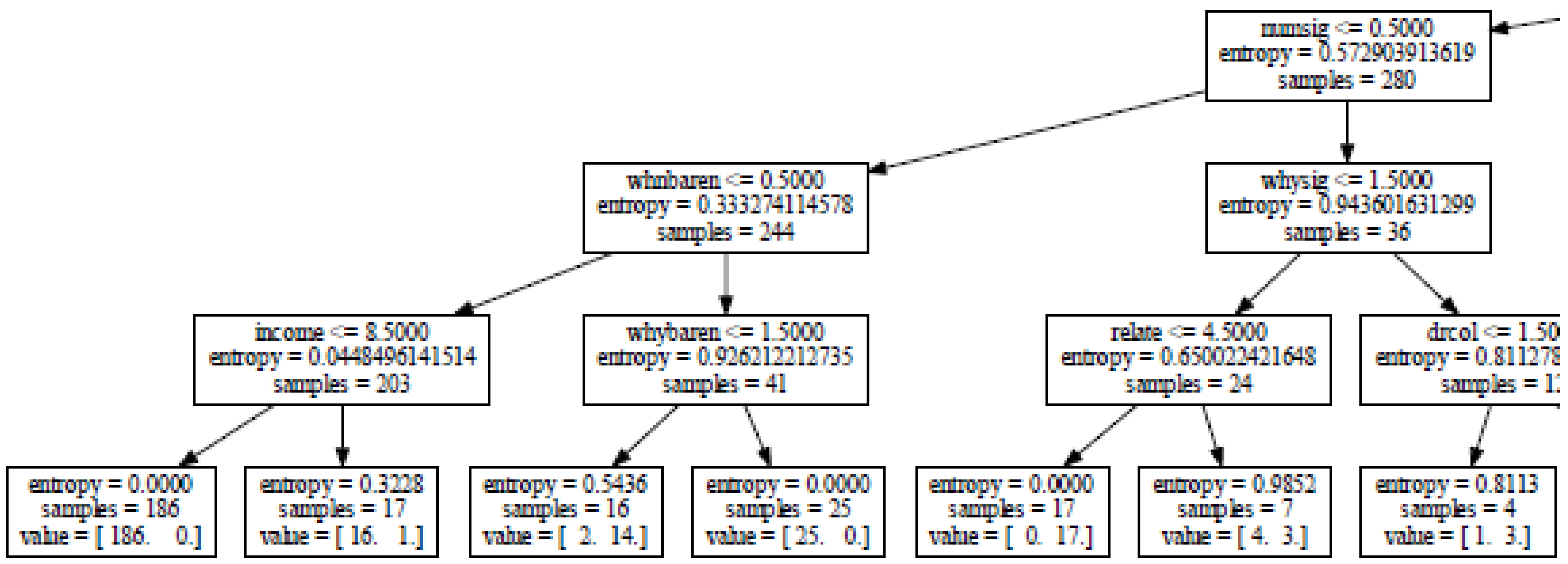
numsig <= 0.5000
entropy = 0.572903913619
samples = 280
whnbaren <= 0.5000
entropy = 0.333274114578
samples = 244
whysig <= 1.5000
entropy = 0.943601631299
samples = 36
income <= 8.5000
entropy = 0.0448496141514
samples = 203
whybaren <= 1.5000
entropy = 0.926212212735
samples = 41
relate <= 4.5000
entropy = 0.650022421648
samples = 24
entropy = 0.0000
samples = 186
value = [186. 0.]
entropy = 0.3228
samples = 17
value = [16. 1.]
entropy = 0.5436
samples = 16
value = [2. 14.]
entropy = 0.0000
samples = 25
value = [25. 0.]
entropy = 0.0000
samples = 17
value = [0. 17.]
entropy = 0.9852
samples = 7
value = [4. 3.]
entropy = 0.8113
samples = 4
value = [1. 3.]

Decision Tree

Depth: 4, Features: All

[Non-adherent, Adherent]

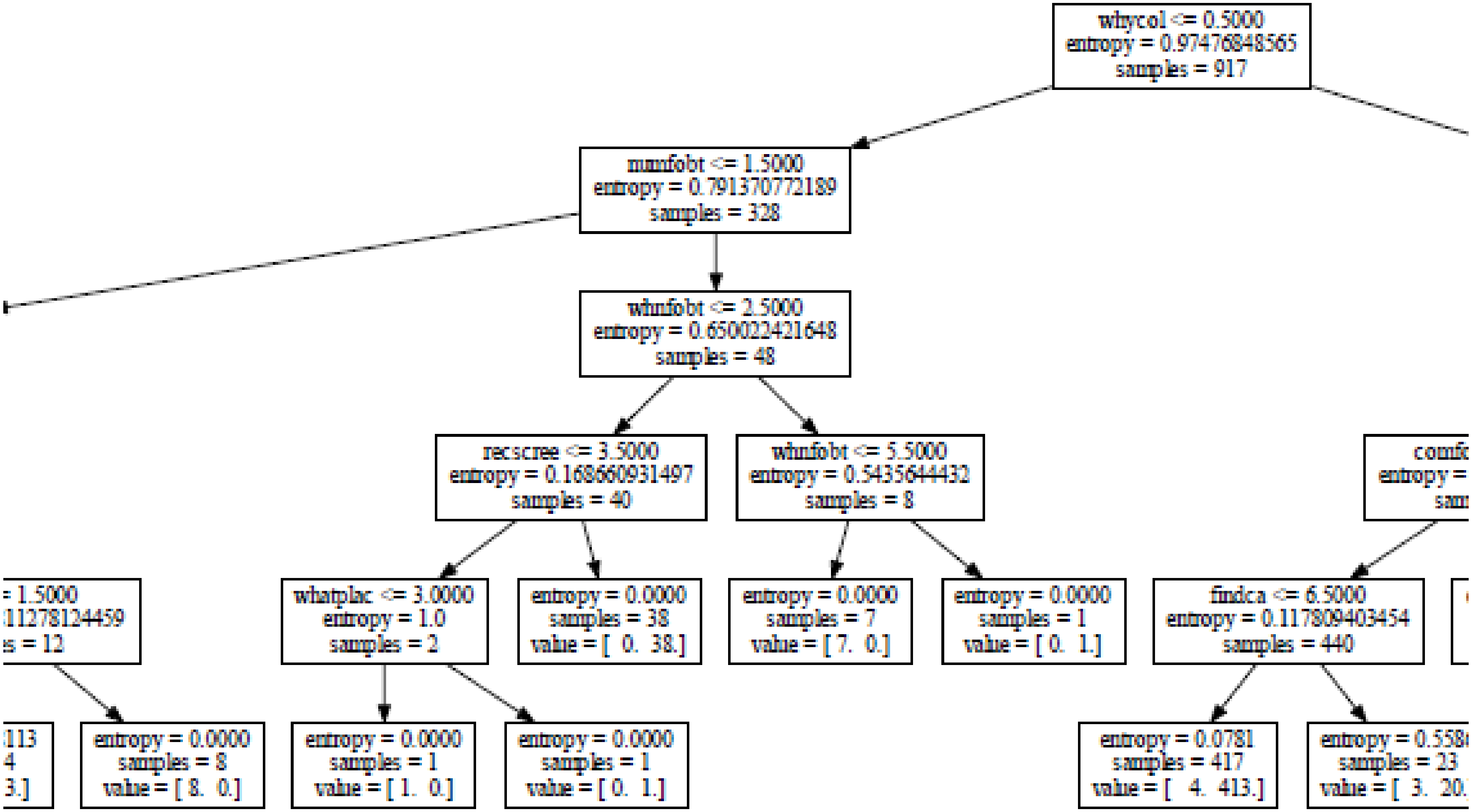

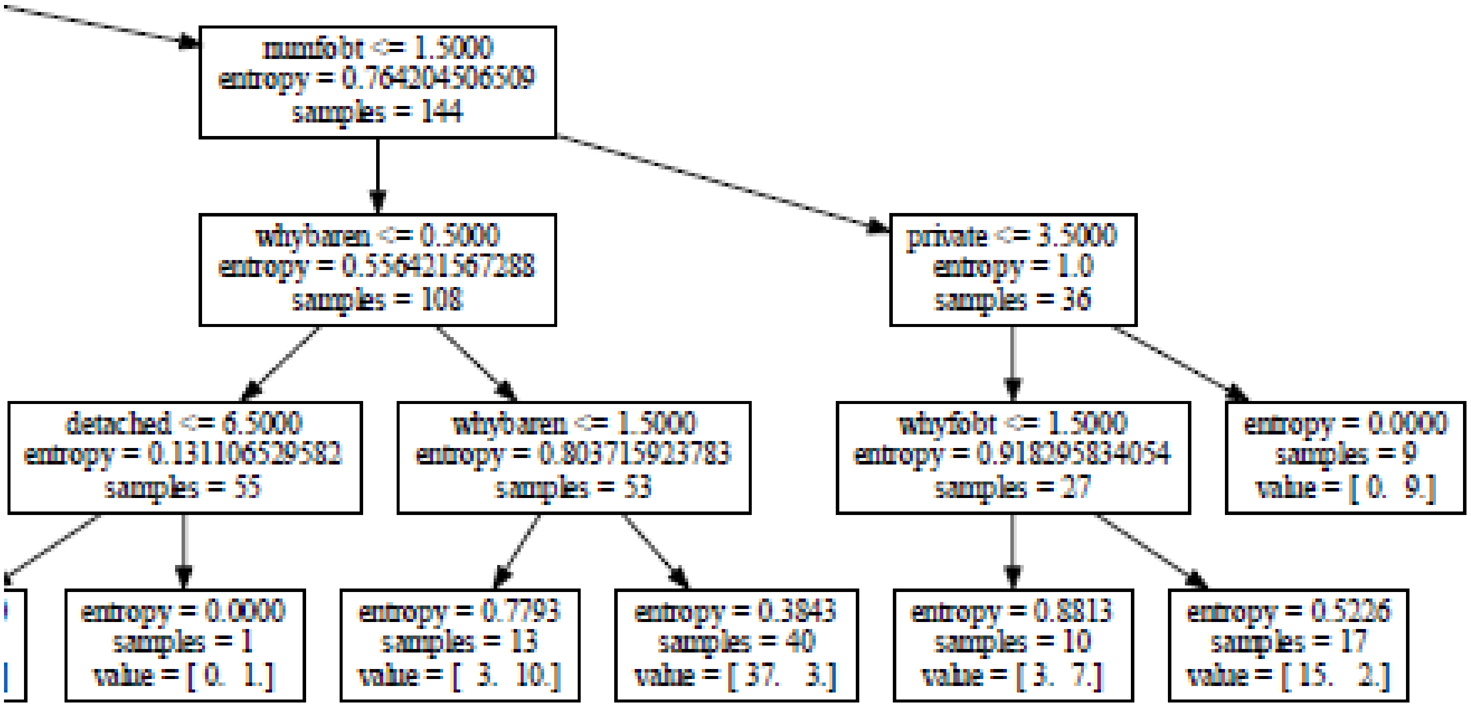
numfobt <= 1.5000
entropy = 0.764204506509
samples = 144
whybaren <= 0.5000
entropy = 0.556421567288
samples = 108
private <= 3.5000
entropy = 1.0
samples = 36
detached <= 6.5000
entropy = 0.131106529582
samples = 55
whybaren <= 1.5000
entropy = 0.803715923783
samples = 53
whyfobt <= 1.5000
entropy = 0.918295834054
samples = 27
entropy = 0.0000
samples = 9
value = [0. 9.]
entropy = 0.0000
samples = 1
value = [0. 1.]
entropy = 0.7793
samples = 13
value = [3. 10.]
entropy = 0.3843
samples = 40
value = [37. 3.]
entropy = 0.8813
samples = 10
value = [3. 7.]
entropy = 0.5226
samples = 17
value = [15. 2.]

Random Forest

In the following table, the **bold** number on the left is the number of features allowed to be used during the tree's creation, and the non-bold number to the right of it is the depth of the tree. Just like the SVM, the random forest was run against all datasets using ten-fold cross validation, with the average accuracy taken for each features, depth combination.

Random forests produced similar results than decision trees, but without the ability to look at a single tree to determine adherence, making it difficult in a clinical setting. It's also harder to tell which variables are more descriptive with a random forest than it is with both decision trees and associative rule mining. As this approach fared worse than its simpler incarnation, decision trees would be the optimal choice in this situation if the choice had to be made.

Features, Depth	Average of Full	Average of Noident	Average of Noclinical
**5**	**0.79845**	**0.78422**	**0.62433**
2	0.7626	0.7291	0.6232
3	0.7703	0.7714	0.6241
4	0.7851	0.7616	0.63
5	0.8106	0.7939	0.6291
6	0.8008	0.7841	0.6468
7	0.8175	0.7949	0.6182
8	0.8067	0.8087	0.6477
9	0.8146	0.791	0.6202
10	0.8106	0.7979	0.63
None	0.8057	0.8096	0.574
**10**	**0.82258**	**0.8208**	**0.64119**
2	0.7832	0.7743	0.6487
3	0.8008	0.7949	0.6389
4	0.8126	0.8145	0.6457
5	0.8175	0.8037	0.6653
6	0.8185	0.8224	0.6418
7	0.8312	0.8352	0.6457
8	0.8263	0.841	0.6516
9	0.838	0.84	0.6378
10	0.841	0.8479	0.6349
None	0.8567	0.8341	0.6015
**15**	**0.84583**	**0.83805**	**0.63979**

2	0.7978	0.7929	0.6418
3	0.8224	0.8057	0.6604
4	0.8194	0.8184	0.6339
5	0.8292	0.8302	0.6349
6	0.8449	0.8321	0.6722
7	0.845	0.8459	0.631
8	0.8489	0.8489	0.6496
9	0.8872	0.842	0.6525
10	0.8734	0.8753	0.6417
None	0.8901	0.8891	0.5799
**20**	**0.85564**	**0.85327**	**0.64523**
2	0.7969	0.7939	0.6497
3	0.8145	0.8057	0.6359
4	0.8253	0.8312	0.6634
5	0.8557	0.8518	0.6526
6	0.8597	0.8714	0.6438
7	0.8803	0.8636	0.6506
8	0.8684	0.8714	0.6565
9	0.8783	0.8793	0.6526
10	0.8843	0.8773	0.6437
None	0.893	0.8871	0.6035
**25**	**0.87684**	**0.87165**	**0.6411**
2	0.8018	0.7959	0.6516
3	0.8263	0.8155	0.6526
4	0.842	0.84	0.6428
5	0.8744	0.8842	0.6349
6	0.8901	0.8783	0.6634
7	0.9009	0.8931	0.6526
8	0.9038	0.9038	0.6477
9	0.9058	0.9127	0.6339
10	0.9146	0.8921	0.6329
None	0.9087	0.9009	0.5986
**30**	**0.87909**	**0.87742**	**0.64414**
2	0.8027	0.8028	0.6448
3	0.8135	0.8292	0.6536
4	0.8577	0.8557	0.6516
5	0.8685	0.8714	0.6379
6	0.9009	0.898	0.6536
7	0.897	0.9048	0.6506
8	0.9165	0.9058	0.6427
9	0.9009	0.8881	0.6535
10	0.9068	0.9107	0.6221

None	0.9264	0.9077	0.631
**50**	**0.90323**	**0.8997**	**0.64436**
2	0.8047	0.8126	0.6507
3	0.8636	0.8734	0.6575
4	0.9058	0.8842	0.6546
5	0.9166	0.9146	0.6516
6	0.9254	0.9234	0.6467
7	0.9166	0.9293	0.6526
8	0.9264	0.9126	0.6251
9	0.9244	0.9117	0.6575
10	0.9352	0.9186	0.6389
None	0.9136	0.9166	0.6084
**75**	**0.92509**	**0.91198**	**0.6484**
2	0.841	0.8391	0.6545
3	0.9156	0.8999	0.6635
4	0.9283	0.9205	0.6507
5	0.9283	0.9225	0.6408
6	0.9401	0.9176	0.6517
7	0.9401	0.9254	0.6644
8	0.9421	0.9264	0.6566
9	0.9332	0.9254	0.6545
10	0.945	0.9254	0.6359
None	0.9372	0.9176	0.6114
**100**	**0.9311**	**0.92098**	**0.64612**
2	0.8862	0.893	0.6605
3	0.9097	0.9087	0.6517
4	0.9313	0.9175	0.6487
5	0.9362	0.9235	0.6496
6	0.9391	0.9254	0.6654
7	0.9421	0.9244	0.6457
8	0.9431	0.9352	0.6526
9	0.9421	0.9284	0.6407
10	0.943	0.9283	0.6339
None	0.9382	0.9254	0.6124
**None**	**0.9365**	**0.92357**	**0.64781**
2	0.896	0.896	0.6605
3	0.9136	0.8999	0.6517
4	0.9372	0.9225	0.6664
5	0.9431	0.9284	0.6585
6	0.948	0.9254	0.6458
7	0.946	0.9323	0.6663
8	0.9558	0.9323	0.6467

9	0.9411	0.9323	0.6369
10	0.948	0.9333	0.6349
None	0.9362	0.9333	0.6104
**Grand Total**	**0.877435**	**0.870164**	**0.642247**

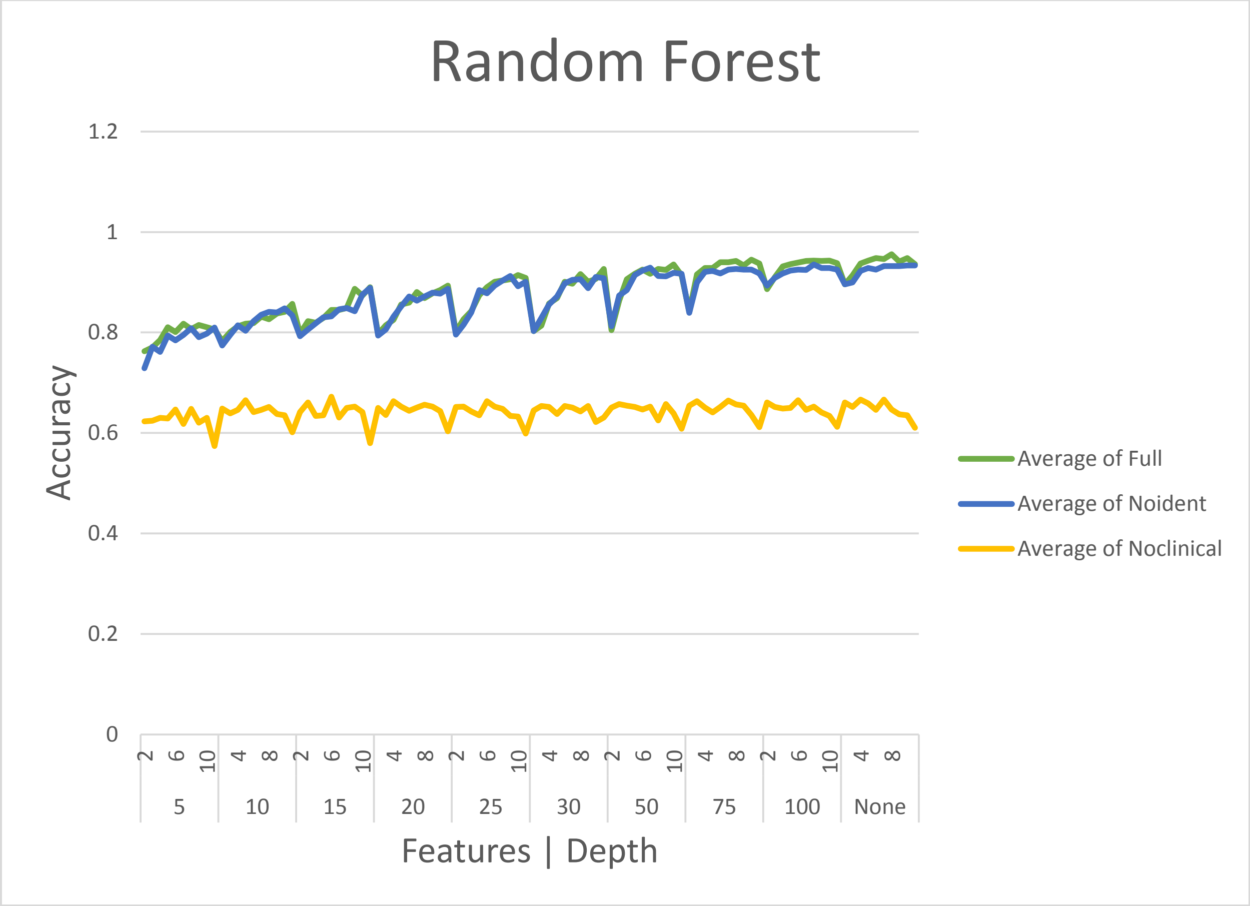

Supplement: Supplementary file 1 — Additional file 1:Results of Test of Data Analytic Methods in Machine Learning and Data Mining.(PDF 601 KB) [file 12889_2014_7327_MOESM1_ESM.pdf]
